# Reverse engineering of metabotropic glutamate receptor-dependent long-term depression in the hippocampus

**DOI:** 10.1186/1471-2202-12-S1-P1

**Published:** 2011-07-18

**Authors:** Tim Tambuyzer, Tariq Ahmed, Daniel Berckmans, Detlef Balschun, Jean-Marie Aerts

**Affiliations:** 1 Department of Biosystems, M3-BIORES: Measure, Model and Manage Bioresponses, Katholieke Universiteit Leuven, Leuven, B-3001, Belgium; 2 Department of Psychology, Laboratory for Biological Psychology, Katholieke Universiteit Leuven, Leuven, 3000, Belgium

## 

This study focused on metabotropic glutamate receptor-dependent long-term depression (mGluR–LTD) in the hippocampus. This form of LTD is suggested to play a key role in learning, memory and the plasticity of behaviour. Recent advances have started to uncover the underlying mechanisms of mGluR-LTD [1]. However, it is not completely clear how these mechanisms are linked and it is believed that several crucial mechanisms still remain to be revealed.

The two main objectives of this study were (i) to quantify the dynamics of mGluR-LTD responses by dynamic data-based models and (ii) to identify underlying dominant processes of mGluR-LTD by applying mathematical system identification methods. In recent years, more and more researchers advocate the use of a top-down modelling approach (reverse engineering) for improving the knowledge of biological systems [2,3].

The drug dihydroxyphenylglycine (DHPG) was used to induce mGluR-LTD in rat brain slices (table 1). The drug was applied for different durations (5min, 15min, 2 hours) and in different concentrations (15mM, 30mM). In addition, also different sampling intervals (5min, 30s, 90s) were used.

**Table 1:** Overview of the experiments

Dataset	DHPG concentration (µM)	Duration of DHPG input	Sampling rate	Age rats	Number of repetitions
1	15	5 min	1/300 s	7-8 weeks	4

2	15	15 min	1/300 s	7-8 weeks	32

3	15	15 min	1/30 s	10-11 months	14

4	30	15 min	1/300 s	8 weeks	10

5	30	15 min	1/30 s	14 months	8

6	30	15 min	1/90 s	15 months	4

7	30	2 h	1/30 s	10-11 months	9

Total number of experiments:					81

For the modelling, discrete-time Transfer Functions (TF) models were used. The models described the relation between the DHPG application (input) and the long-term depression responses (output).

All models were very accurate (all R_T_^2^-values higher than 0,94) and reliably estimated. For a 2 hours application of 30 µM DHPG sampled with a frequency of 1/30s, the time-constant of the mGluR-LTD response was 92s. Thus, the models for high sampling rate indicated that a sampling interval of 30s would be ideal to minimize information loss of the dynamics of mGluR-LTD responses.

Interestingly, it was suggested that there are three dominant sub-processes underlying mGluR-LTD: one fast sub-process, one slow sub-process and an immediate sub-process.

This study suggests that the dynamic data-based modelling approach can be a valuable tool for reverse engineering of mGluR-dependent LTD responses.
